# A look into the virosphere of clouds: A world yet to be explored

**DOI:** 10.1016/j.crmicr.2025.100545

**Published:** 2025-12-31

**Authors:** Janina Rahlff, Pierre Amato

**Affiliations:** a Centre for Ecology and Evolution in Microbial Model Systems (EEMiS), Department of Biology and Environmental Science, Linnaeus University, Kalmar, Sweden; b Aero-Aquatic Virus Research Group, Faculty of Mathematics and Computer Science, Friedrich Schiller University Jena, Jena, Germany; c Leibniz Institute on Aging - Fritz Lipmann Institute (FLI), Jena, Germany; d Université Clermont Auvergne, CNRS, Laboratoire Microorganismes: Génome et Environnement, UMR 6023, F-63000 Clermont-ferrand, France

**Keywords:** Atmosphere, Virus, Phage, Bacteria

## Abstract

Clouds are aqueous atmospheric systems hosting diverse and active microorganisms. Viruses may also persist despite harsh conditions, support active viral infections, and contribute to microbial dynamics during aerial transport. However, assessing viruses, and even more, virus-bacteria interactions in the atmospheric environment is highly challenging, and knowledge remains very limited. Here, based on current knowledge in cloud microbiology, we estimate the cloud virome at ∼10^21^ virus particles globally. One out of a million of cloud droplets is susceptible to host virus-bacteria interactions, which represents considerable volume at global scale, and we discuss potential implications for microbial ecology. We finally propose future research directions to explore further the cloud virosphere and its ecological roles.

## Highlights

• Viruses are largely overlooked in outdoor aeromicrobiological studies.
• Based on current knowledge in aeromicrobiology, a total of 10^21^ virus particles are estimated to occupy clouds globally.
• Although very dilute, these may contribute to the atmospheric life cycle of microorganisms.
• Virus-bacteria interactions in clouds would have many implications in ecology and the Earth’s microbiome.

## Graphical abstract

**Figure:**
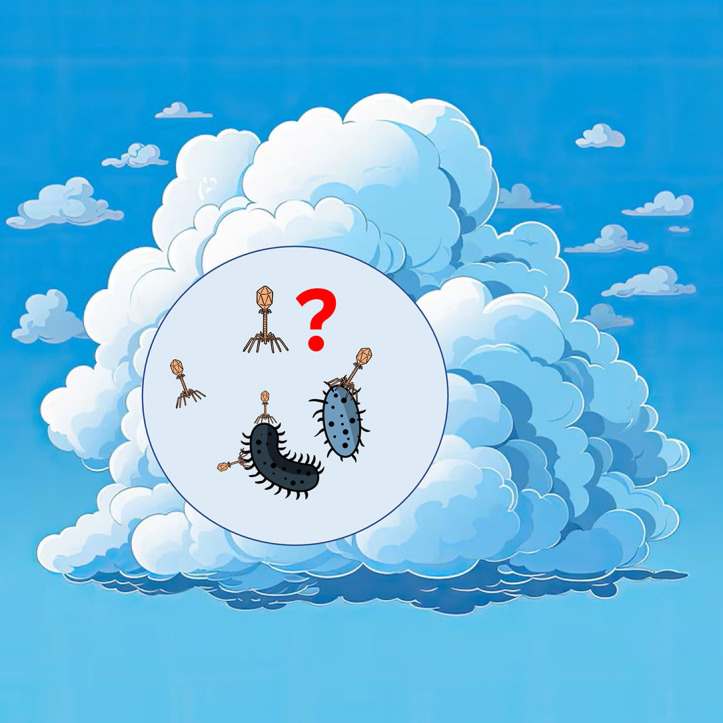

## Clouds as carriers for active microbes

Near surfaces, viruses circulate in the air at concentrations around 10^6^ to 10^7^ m^-3^, with identified variations linked with seasons (e.g., Whon et al. (2012)). Concentrations are expected to decrease with increasing elevation above ground, as inferred from studies on airborne microbiomes (Drautz-Moses et al., 2022; Rodó et al., 2024), although a fraction of particles may still reach cloud altitudes.

A cloud is a visible volume of water droplets or ice crystals suspended in the atmosphere, at typical concentration of ∼100 droplets per cm^-3^, with a size of ∼10 µm in diameter, resulting in a total liquid water content of ∼0.3 g (or mL) of water m^-3^ of cloud volume (Miles et al., 2000). These form when moist air rises and cools, causing water vapor condensation at the surface of aerosol particles. At the global scale, clouds represent ∼15 % of the total tropospheric volume (Pruppacher and Jaenicke, 1995).

Microbes in clouds consist of highly diverse taxa of bacteria, fungi and others resulting from the mixing of multiple influencing sources (Barberán et al., 2015; Fierer et al., 2008; Tignat-Perrier et al., 2019). In bacteria, frequent viable genera identified from cultures or molecular studies include Pseudomonadota, such as *Pseudomonas, Sphingomonas, Methylobacterium*, Actinomycetota such as *Rhodococcus*, and Bacillota, such as *Bacillus* and *Staphylococcus* (Amato et al., 2017; Vaïtilingom et al., 2012a).

We assume that the microbial cohorts from surface ecosystems reaching clouds also include at least part of their viromes, either within cells, or as free-living viral particles. Numerous studies notably pointed out the prevalence of plant-associated microorganisms (bacteria, fungi) in clouds, but phyllosphere’s and other viral communities and their members remain poorly known (Sharma et al., 2021). Given the large surface area, oceans and seas also likely represent strong sources of airborne viruses at the global scale (Aller et al., 2005; Alsante et al., 2021; Baylor et al., 1977). In the atmosphere of oceans, airborne viruses can also be dust-associated (Yahya et al., 2019).

From a microbial ecology perspective, clouds have been regarded as temporary aquatic habitats for airborne microbes, providing microdroplets of water that sustain microbial survival and activity for limited time (minutes to hours) before removal by precipitation or sedimentation (Amato, 2012; Péguilhan et al., 2025). As such, in addition of acting as vehicles for the transfer of microorganisms across different ecosystems, clouds and fogs provide airborne viable microorganisms with condensed water and dissolved nutrients, i.e., conditions compatible with metabolic activity and possibly cell multiplication (Amato et al., 2007; Fuzzi et al., 1997; Sattler et al., 2001). Metatranscriptomics studies indicate that bacteria and eukaryotes exhibit multiple biological functions in clouds, largely oriented toward the response to stresses: oxidants, osmotic shocks, temperature shifts and nutrient limitations (Amato et al., 2019; Péguilhan et al., 2025); the metabolic pathways potentially involved were further specified through metabolomics (Jarrige et al., 2025; Jousse et al., 2018; Wirgot et al., 2019). In the water of clouds, heterotrophic bacteria have been estimated to process up to ∼10 million tons of organic carbon every year from small dissolved organic compounds such as formate, acetate, and formaldehyde (Ervens and Amato, 2020; Vaïtilingom et al., 2012b).

With an average of ∼10^4^ cells mL^-1^ of cloud water (Vaïtilingom et al., 2012a), as usually quantified in clouds at the mid-altitude mountain observatory ‘puy de Dôme’ (1465 m asl, central France), we estimate that, conservatively considering 1/10th this concentration as a global average, a total of ∼10^20^ bacteria occupy clouds on a global scale (half of this number was reported for the atmospheric boundary layer (Šantl-Temkiv et al., 2022; Whitman et al., 1998)). Considering a virus-to-host ratio of 10:1 as reported in marine environments (Suttle, 2005), we therefore expect the global cloud virome to represent ∼10^21^ viruses in total, at an average concentration of ∼10^5^ viruses mL^-1^. The virus-to-bacteria ratio in outdoor air has been rarely measured, with one value reported of 1.4 based on sampling on a university campus and staining virus particles on a filter (Prussin et al., 2015) and other measurements varying widely between 0.01 and 10,000 (Prussin et al., 2014). A ratio of 10:1 virus-to-bacteria therefore appears reasonable, keeping in mind the high level of uncertainty and expected spatial and temporal heterogeneity associated with this value. At the high levels of mixing present in the atmosphere, viruses may encounter new hosts in clouds that they would otherwise be physically separated from in their natural habitats. This could greatly accelerate and expand the interspecific horizontal transfer of genetic material. In the atmosphere, viruses can exist in particles and small droplets that are easily dispersed (Reche et al., 2018). As for bacteria, and even more due to smaller size, viral dispersal via atmospheric transport may reach intercontinental scales (Smith et al., 2013).

To date, few studies have reported on the existence of different types of viruses in clouds (Amato et al., 2019; Castello, 1995), fog, precipitation and outdoor air (Amato et al., 2019; Castello, 1995; Das et al., 2025; Deng et al., 2024; Jiang et al., 2025; Rahlff et al., 2023; Whon et al., 2012). Virus numbers in air have been considered negligible (Mushegian and Margolin, 2020), although estimates of the upper limit of viral concentration in ambient air reach 3 × 10^4^ m^−3^ (Després et al., 2012) and reports for airborne dust range from 7 × 10^4^ to 3.1 × 10^6^ viral particles m^−3^ (Yahya et al., 2019). Airborne viruses deposit on surfaces at rates of ∼10^9^ m^−2^ d^-1^ in pristine high-altitude environment, i.e.*,* ∼100 times higher than bacteria, in line with the deposition of submicron organic aerosols (<0.7 µm) (Reche et al., 2018). Virus-particle counts from rainwater (originating from clouds) sampled at a Swedish coast close to sea level, have ranged between 10^4^ and 10^5^ viral-like particles per mL of rainwater (Rahlff et al., 2023). In the free troposphere at a high-altitude site, the deposition rates of viruses from the atmosphere to the surface reached ∼10^8^ to ∼10^10^ virus m^-2^ d^-1^, i.e., 9 to 461 times greater than that of bacteria cell number (Reche et al., 2018). We assume that viral concentrations vary with multiple factors, including sampling location, environmental context (e.g., air pollution, season, ground vegetation, or weather conditions), and the sampling method employed (e.g., impingers versus dry collectors).

## Viral-bacterial interactions could reshape our understanding of clouds as ecosystems

There is no evidence yet that viable viruses are circulated at large scale with clouds, and if they could contribute to regulate microbial diversity and to the transfer of genetic material during aerial transport. The current vision of clouds as biotic systems is that microorganisms, due to low biomass and distribution in micro-droplets, cannot or are unlikely to interact with each other in these environments: statistically, only one out of >10,000 cloud droplets is expected to contain a bacterial cell (i.e., droplets are 10,000 times more numerous than bacteria in a given volume) (Ervens and Amato, 2020; Ervens et al., 2025; Fankhauser et al., 2019). However, the plausibility of microbial interactions in clouds has yet to be evaluated experimentally. Notably, identifying bacteriophages (phages) interacting with bacteria cells in clouds would constitute an important step forward in our understanding of the influence of these environments on microbial life cycles.

The rapid advent of molecular methods (metagenomics) makes it possible to identify viral sequences in clouds (Péguilhan et al., 2025). A decisive step further in the description of cloud’s viromes would consist in evidencing the maintenance of their integrity, viability, and infectivity, and ultimately detecting, if any, on-going lytic processes in cloud droplets, through markers such as presence of structural, assembly genes, or lysis genes. Absence of integrases or repressor genes on viral genomes would further support lytic behavior as those genes typically maintain lysogeny, a hallmark of temperate phages integrated into the host chromosome. Existing (meta)genomic resources from cloud water microbial studies (Amato et al., 2019; Besaury et al., 2017; Jarrige et al., 2022; Lallement et al., 2017; Péguilhan et al., 2025) could be used to screen for prophages. If prophages are identified in metagenome-assembled genomes or in genomes of prokaryotic isolates from cloud water, induction assays combined with microscopy could determine whether they can transition to the lytic cycle, in which the phage destroys the host cell to be released. The atmosphere indeed selects for prophage carrying microbes, and these prophages are often inducible (Teigell-Perez et al., 2019).

Models have been proposed to evaluate the risks associated with airborne viruses and their survival under atmospheric conditions (Casal, 1995; Hofmeister et al., 2021). However, important aspects such as viral ecology, lifestyle prevalence, diversity, variant formation (viral microdiversity), and host interactions (including antiviral defense systems) are missing in our knowledge on the cloud water and most air ecosystems. Previous work has shown that airborne viral communities are habitat-specific - including the auxiliary metabolic genes they carry -, comprise diverse viral groups (e.g., dsDNA *Caudoviricetes,* ssDNA *Repensiviricetes,* and Nucleocytoviricota), which include animal and plant pathogenic viruses, and are influenced by both sampling height and predicted source (Das et al., 2025; Deng et al., 2024; Jiang et al., 2025; Rahlff et al., 2023; Whon et al., 2012). Another feature of DNA airborne viruses seems to be a high G + C base content of their genome (Das et al., 2025; Rahlff et al., 2023) potentially reflecting resistance to environmental stress and UV radiation. Despite the low concentration of viruses in the atmosphere, a large number of previously unknown viruses is suspected to be present (Whon et al., 2012).

Our understanding of viruses in atmospheric contexts is currently limited, in part due to missing sequence data on airborne viruses available from reference databases such as IMG/VR (Camargo et al., 2023) and its successor MetaVR (Fiamenghi et al., 2025) with only 639 UViGS derived from outdoor air ecosystems at the time of writing. In addition, the general focus is on performing research on viruses relevant to humans (Rodrigues et al., 2017), for instance in epidemiological context to infer the airborne spread of viral infections (Sattar and Ijaz, 1987). Enhanced by the SARS CoV-2 virus pandemic comes a focus on (indoor) air sampling of pathogenic viruses related to the transmission of infectious diseases and the technology needed to conduct such samplings (Chang et al., 2023; Wei et al., 2024). Air samplers may fail to collect viruses freely suspended in very small droplets due to limitations in air sampling techniques, which typically capture aerosol particles > 500 nm (Zhang et al., 2022), which is larger than most viruses. As a result, -omics approaches might overestimate the prevalence of certain airborne viruses, such as larger viruses (e.g., giant viruses) or viruses attached to hosts or larger particles, while missing others.

## Challenges of host encounter and infection for airborne viruses

As a result of atmospheric transport, viruses may end up in environments quite different from where they started, potentially affecting their ability to find and infect their preferred host cells (Prussin et al., 2014). Viruses vary widely in host specificity: while some infect only a single bacterial strain (Holmfeldt et al., 2007) many others have broad host ranges and can infect multiple hosts (Bignaud et al., 2025). Consequently, the likelihood of infection may be limited for particular host cells depending on host-virus compatibility. In the cloud environment, airborne viruses may face significant challenges in locating a host, as their replication depends on hijacking a host cell’s machinery, a process that likely becomes more complex for viruses in the hostile and extreme environment of the cloud. We assume that in the atmospheric environment the likelihood for the establishment of microbial associations is generally rare (Ervens et al., 2025). Therefore, the probability for viruses and cells encountering each other diminishes due to the dispersed nature of viral particles and the specific environmental conditions. When considering clouds, which often have very low temperatures far below zero degree, but can be supercooled down to −37.5 °C (Rosenfeld and Woodley, 2000), the likelihood of viruses encountering a host and successfully replicating becomes even more uncertain. Freeze-thaw cycles, which are likely to act on viruses in clouds, can harm the infectious particles to the point of rendering them non-active or resulting in loss of tails in bacteriophages (Cox et al., 1974). The complexity and combination of environmental processes within clouds could also affect crucial stages of the viral replication cycle, such as the adsorption phase, e.g.*,* by altering the stability and behavior of viral particles. Previous experiments with cold-active bacteriophages of the host *Shewanella* from the Baltic Sea have shown that adsorption rates of two phages at 4 °C and 15 °C were like those of phages infecting at mesophilic conditions (Sencilo et al., 2015). However, it remains to be elucidated whether the combined burden of intermittent freezing, UV stress, and osmotic shocks, and other conditions characteristic of the cloud water habitat would lead to different effects. The temporal dynamics in particular are rarely explored throughout such short temporal scales. In the Arctic Ocean, higher lysogeny rates occur at low temperatures and under oligotrophic conditions, when productivity is lowest (Payet and Suttle, 2013). These observations could be transposed to clouds, although the temporal dynamics strongly differ. High lysogeny rates in clouds would imply high level of genome reorganization in microbial cells, and, along with the mutations potentially caused by elevated UV exposure, would position clouds as potential ‘reactors’ of microbial evolution (i.e., environments prone to evolution and functional innovation).

If viruses in clouds possess specific adaptations such as reversible inactivation, capsid robustness in face of different stress (Coleman et al., 2024; Martín-González et al., 2018) or association with microbial aggregates during dispersal or transitions between hosts, requires future investigation. Desiccation tolerance-such as through silicification (i.e., coating in silica) (Laidler et al., 2013; Laidler and Stedman, 2010) could significantly enhance viral survival and transmission in the atmosphere. Silicification is unlikely to occur in cloud water itself. However, pre-existing mineral associations acquired before aerosolization could play a role in atmospheric virus stability. Viability of bacteria suspended in aerosols decreased with decreasing relative humidity (RH), whereas viruses showed most reduced viability at intermediate RH (Lin and Marr, 2020). Because clouds occur at RH ≥100 %, conditions may favor the persistence of both bacteria and viruses. While non-lipid viruses (viruses lacking a lipid envelope, such as bovine adenovirus type 1) remain stable at high RH, most lipid-containing viruses (enveloped viruses with a lipid membrane, such as equine arteritis virus and vesicular stomatitis virus) are highly sensitive and are therefore not expected to persist or maintain infectivity in clouds (Donaldson and Ferris, 1976). Evaporation-induced pH shifts may drive conformational changes in the surface glycoproteins of enveloped viruses, leading to altered infectivity (Yang and Marr Linsey, 2012). Similarly, work by Harper (1961) demonstrated that temperature and RH have distinct as well as interactive effects on airborne viral viability (“survival”), with lower temperatures generally enhancing viability, while the effects of RH are virus-dependent and complex (Harper, 1961; Longest et al., 2024), which has also been shown for phages (Verreault et al., 2015). Effects of UV-C radiation on airborne phages and their infectivity was also phage-dependent, with the ssRNA phage MS2 infecting *Escherichia coli* being most resistant (Verreault et al., 2015). Together, these findings suggest that cloud environments, which are characterized by fluctuating temperature, RH, pH, and radiation, can differentially modulate viral viability and infectivity, implying that clouds may act as selective and dynamic reservoirs for airborne viruses rather than passive transport media.

## How likely are viral infections in cloud water?

**Fig. 1:**
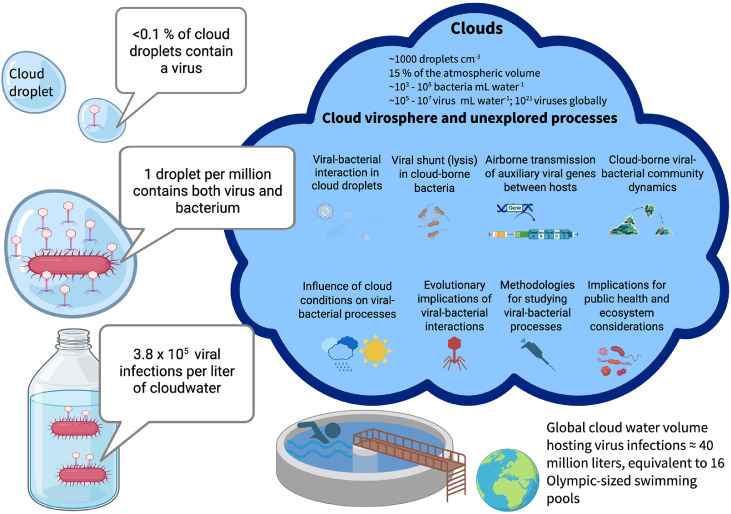
Processes and open research topics in the cloud virosphere. Created in BioRender. Rahlff, J. (2025)https://BioRender.com/xol958f.

Bacterial concentrations range from 10³ to 10⁵ cells mL^-1^ in cloud water (Nuñez López et al., 2024). Assuming a spherical droplet with diameter 10 µm (volume ≈ 5.24 × 10^−13^ L, approximately 5.24 × 10^−10^ mL, typical for stratus clouds (Miles et al., 2000; Wallace and Hobbs, 2006)), this corresponds to roughly one bacterial cell every ∼1.9 × 10^4^ to ∼ 1.9 × 10^6^ droplets (equivalent to ∼5.2 × 10^–7^ to ∼5.2 × 10^–5^ bacteria cell droplet^-1^). As a conservative estimate, we therefore consider that 1 out of ∼10^6^ droplets harbor a bacteria cell. From this, we estimate one virus to occur every ∼ 1.9 × 10^3^ to ∼ 1.9 × 10^5^ droplets (i.e., ∼5.2 × 10^–6^ to ∼5.2 × 10^–4^ virus droplet^-1^) assuming a virus-to-host ratio of 10:1 as observed in marine environments (Suttle, 2005). Viruses and bacteria are likely collocated in cloud droplets (Fig. 1), as they are frequently co-emitted from common sources such as plant surfaces, soils, and ocean spray (Aller et al., 2005; Banttari and Venette, 2006; Girardin et al., 2016). A conservative estimate is therefore that one in every 10^6^ cloud droplets, contains at least one virus and one bacterium in collocation; this value logically corresponds to the conservative estimate for the frequency of bacteria. Assuming a conservative infection success probability of 20 %, consistent with lower-bound estimates for phage infection efficiency in marine systems (Suttle, 2005), the expected infection rate is ≈ 0.2 successful infections per 10^6^ droplets. Due to the lack of cloud-specific data, we use a well-established value for viral infection success derived from marine systems, while acknowledging that cloud conditions may lead to different outcomes. Scaled up to bulk water, this corresponds to 3.8 × 10^5^ infections per liter of cloud water (≈ 382 infections per mL). At the global scale, considering a total mass of condensed water of 2 × 10^17^ g (Pruppacher and Jaenicke, 1995), and the average cloud droplet size of 10 µm (Wallace and Hobbs, 2006), this corresponds to an estimated total of 3.8 × 10^26^ cloud droplets globally (water density of 1 g mL^-1^), of which then 7.5 × 10^19^ are expected to contain viral-bacterial infections. These latter therefore occur at very high concentration (equivalent to literally >10^9^ virus-bacteria pairs per mL within the water droplets concerned), in a total volume of 40 million liters at the global scale, equivalent to 16 Olympic-sized swimming pools. Although the infection probability in each cloud droplet is low and may seem insignificant, the size of the atmosphere and the vast number of droplets at the global scale implies that viral infections in cloud environments could be ecologically significant, potentially influencing microbial dynamics and biogeochemical processes on a planetary scale. However, this estimate is based on different assumptions and considers averages of current knowledge in aeromicrobiology. This does not account for environmental variability and dynamics, or host-virus specificity. In addition, air sampling methods may bias the recovered viral population depending on sampler design and viral size, and post-collection artifacts may influence observed virus-host associations.

After being released from a host cell, the fate and potential further replication of viruses in the atmosphere are subject to various factors including radiation exposure. Higher levels of radiation can impact mutation rates of viral nucleic acids and may lead to a selection of genomes featuring high proportion of the guanine cytosine (GC) content in both viruses and bacteria (Ellington et al., 2021; Rahlff et al., 2023). Viruses facilitate horizontal gene transfer among microbes, which can lead to the acquisition of new traits and the evolution of microbial communities. This genetic exchange occurs through processes such as transduction, where viral particles accidentally package and transfer host DNA to new cells. Transduction can introduce novel genes into microbial populations, potentially conferring advantages such as benefitting cold survival (Rahlff et al., 2024) photosynthesis (Sullivan et al., 2006), or withstanding UV-mediated DNA damage. Via aerosols, clouds, and precipitation and therein residing viruses these genes can easily spread into new environments. Airborne viromes were not found to carry antibiotic resistance genes yet (Maestre‐Carballa et al., 2024) but they are key carriers of these genes in many environments (Debroas and Siguret, 2019). Instead, different metabolically relevant genes have been found (Deng et al., 2024). By manipulating host cell metabolism and subsequent lysis, viruses could release cellular contents into the cloud environment, which can be taken up by other microorganisms. This “viral shunt”-like process could contribute to nutrient cycling within cloud ecosystems creating ecological niches for less common or new microorganisms, and contribute to the diversity and atmospheric dispersal of the various microbial species that are found in clouds (Péguilhan et al., 2023a) .

## Future research needs and implications

Clarifying the actual number concentration and distribution of viruses in cloud water would help to establish baselines for viral activity in clouds and impacts on cloud microbial ecology. Nevertheless, to improve our knowledge, there is a pressing need for advanced air sampling methods that can effectively collect airborne material small enough to include (single) viruses. In addition, as viruses likely represent a low proportion of the airborne biological material, powerful sampling methods are required to overcome technical limitations and yield sufficient amounts of nucleic acids for the analysis (Péguilhan et al., 2025, 2023b; Santl-Temkiv et al., 2017). Concentration-based approaches such as ultrafiltration or chemical flocculation commonly used for aquatic samples (Langenfeld et al., 2021) may be required to increase recoverable material for downstream analyses. For example, the low biomass of cloud water could be mitigated by co-flocculating bacterial and viral particles (Rahlff et al., 2025). Microscopy-based visualization (epifluorescence or electron microscopy) could provide an independent means of validating viral presence and assessing particle integrity following cloud water processing.

Additionally, we have limited knowledge on how viral abundance, diversity, and stability vary with the dynamic factors that influence cloud formation and atmospheric conditions such as temperature, water availability, condensation/evaporation cycles, UV light, aerosol, and gas chemical composition. Another challenge is that viral replication and phage-host encounters may occur within the sampling bottle itself, where particles or droplets are accumulated and collected, potentially altering the native composition and interaction dynamics of the cloud water microbial assemblage. This raises concerns about post-sampling artifacts, where conditions in the collection vessel such as temperature shifts or prolonged incubation could enable interactions that would not occur in the atmosphere. Careful handling protocols and time-resolved sampling may be necessary to distinguish true in situ events from those arising during sample storage or processing.

To complement short-term interaction studies, it may be useful to investigate both CRISPR-based phage-host interactions and viral genomic variants, as they reflect longer-term evolutionary dynamics that are unlikely to emerge during or shortly after sample collection. CRISPR (Clustered Regularly Interspaced Short Palindromic Repeats) is an adaptive immune system in bacteria and archaea that stores sequences of past viral invaders as “spacers” within CRISPR arrays. When a previously encountered virus infects the cell again, these spacers guide CRISPR-associated Cas proteins to recognize and neutralize the invader. CRISPR spacer acquisition provides a genomic record of past phage exposure and defense (Barrangou and Horvath, 2009), offering insights into the co-evolutionary history of microbial communities in cloud water.

Once it is determined whether viruses can replicate in clouds, it would make sense to explore whether a hypothetical 'Tinker Bell virus' could stay airborne indefinitely. This virus would always need to infect new airborne hosts, like the recently proposed 'Peter Pan microbe,' which is said to 'never land' (Tastassa et al., 2024). During our investigation of cloud water bacterial communities, we observed that certain strains readily form extensive aggregates in liquid culture and rapidly (within few hours) establish biofilms on the surfaces of well plates (unpublished observation). Such multicellular structures may facilitate viral propagation by enabling direct or short-range transfer between neighboring bacterial cells, especially facilitating infections of temperate phages (Winans et al., 2025). Phages may also inhibit aggregate formation and dissemination (Darch et al., 2017). To test these hypotheses and better understand phage-host interactions in atmospheric environments, the development of a model phage-host system derived from cloud water isolates would be highly valuable. Crucially, these interactions should be studied under simulated atmospheric conditions, such as low temperature, high UV exposure, and dynamic humidity to realistically assess the ecological relevance and feasibility of viral infections in cloud microenvironments.

## Conclusions

In conclusion, many questions remain to be answered about the presence of viruses in clouds. Key uncertainties include the concentration, diversity, abundance and prevalence of viruses in cloud water, how environmental conditions influence viral persistence, replication, structure, diversity and the impact of viruses on cloud microbial dynamics and nutrient cycling. Addressing these gaps requires advanced sampling methods, detailed ecological studies, and an interdisciplinary approach to understand how viruses interact with microbial communities in the sky. As we advance our knowledge in this area by combining collaborative forces, we may uncover new insights into the complex ecological roles of viruses in atmospheric systems and their broader implications for microbial life and environmental processes.

**List of abbreviations**
CRISPR: Clustered Regularly Interspaced Short Palindromic Repeats
rRNA: ribosomal RNA
SARS-CoV-2: Severe Acute Respiratory Syndrome Coronavirus
UV: Ultraviolet
UViGS: Uncultivated Virus Genomes
RH: Relative humidity

## Ethics approval and consent to participate

Not applicable.

## Consent for publication

A publication license from BioRender for the figure is available.

## Availability of data and materials

Not applicable.

## Funding

JR received funding by the Swedish Research Council, Starting Grant ID 2023–03310_VR and a Procope Mobility 2024 grant from the Department for Science and Technology of the Embassy of France in Germany.

## Authors' contributions

JR and PA contributed equally to calculations, writing, and visualization.

## Declaration of competing interest

The authors declare that they have no known competing financial interests or personal relationships that could have appeared to influence the work reported in this paper.
